# A Joint-Parameter Estimation and Bayesian Reconstruction Approach to Low-Dose CT †

**DOI:** 10.3390/s23031374

**Published:** 2023-01-26

**Authors:** Yongfeng Gao, Siming Lu, Yongyi Shi, Shaojie Chang, Hao Zhang, Wei Hou, Lihong Li, Zhengrong Liang

**Affiliations:** 1 Department of Radiology, Stony Brook University, Stony Brook, NY 11794, USA; 2 Department of Medical Physics, Memorial Sloan Kettering Cancer Center, New York, NY 10065, USA; 3 Department of Preventive Medicine, Stony Brook University, Stony Brook, NY 11794, USA; 4 Department of Engineering Science and Physics, CUNY/CSI, Staten Island, NY 10314, USA

**Keywords:** low-dose CT, Bayesian reconstruction, hyperparameter, probability density function

## Abstract

Most penalized maximum likelihood methods for tomographic image reconstruction based on Bayes’ law include a freely adjustable hyperparameter to balance the data fidelity term and the prior/penalty term for a specific noise–resolution tradeoff. The hyperparameter is determined empirically via a trial-and-error fashion in many applications, which then selects the optimal result from multiple iterative reconstructions. These penalized methods are not only time-consuming by their iterative nature, but also require manual adjustment. This study aims to investigate a theory-based strategy for Bayesian image reconstruction without a freely adjustable hyperparameter, to substantially save time and computational resources. The Bayesian image reconstruction problem is formulated by two probability density functions (PDFs), one for the data fidelity term and the other for the prior term. When formulating these PDFs, we introduce two parameters. While these two parameters ensure the PDFs completely describe the data and prior terms, they cannot be determined by the acquired data; thus, they are called complete but unobservable parameters. Estimating these two parameters becomes possible under the conditional expectation and maximization for the image reconstruction, given the acquired data and the PDFs. This leads to an iterative algorithm, which jointly estimates the two parameters and computes the to-be reconstructed image by maximizing a posteriori probability, denoted as joint-parameter-Bayes. In addition to the theoretical formulation, comprehensive simulation experiments are performed to analyze the stopping criterion of the iterative joint-parameter-Bayes method. Finally, given the data, an optimal reconstruction is obtained without any freely adjustable hyperparameter by satisfying the PDF condition for both the data likelihood and the prior probability, and by satisfying the stopping criterion. Moreover, the stability of joint-parameter-Bayes is investigated through factors such as initialization, the PDF specification, and renormalization in an iterative manner. Both phantom simulation and clinical patient data results show that joint-parameter-Bayes can provide comparable reconstructed image quality compared to the conventional methods, but with much less reconstruction time. To see the response of the algorithm to different types of noise, three common noise models are introduced to the simulation data, including white Gaussian noise to post-log sinogram data, Poisson-like signal-dependent noise to post-log sinogram data and Poisson noise to the pre-log transmission data. The experimental outcomes of the white Gaussian noise reveal that the two parameters estimated by the joint-parameter-Bayes method agree well with simulations. It is observed that the parameter introduced to satisfy the prior’s PDF is more sensitive to stopping the iteration process for all three noise models. A stability investigation showed that the initial image by filtered back projection is very robust. Clinical patient data demonstrated the effectiveness of the proposed joint-parameter-Bayes and stopping criterion.

## 1. Introduction

Tomography, as one non-invasive imaging technique, has a wide application in many scientific fields, e.g., physics, chemistry, astronomy, medicine, etc. [1]. There are a variety of tomographic modalities, such as computed tomography (CT), nuclear medicine, ultrasound diffraction tomography and so on. Under certain conditions, the measurements in each modality can be converted into samples of the Radon transform distribution from which we wish to reconstruct the corresponding image [2]. The inversion of Radon transform is well-established by deterministic methods: for example, the filtered back-projection (FBP) [3]. However, there are many scenarios in which the measurements are incomplete data, which will significantly degrade the performance of the deterministic method. For instance, noise artifacts will appear in low-dose CT imaging due to the incomplete data. In such a scenario, the Bayesian theorem-based penalized maximum likelihood methods [4], which incorporate the data fidelity and prior knowledge into one unified objective function, provide an alternative way to deal with the imperfect data acquisitions.

Most penalized maximum likelihood reconstruction methods include one freely adjustable hyperparameter to balance the strength between data fidelity and prior terms. Specifically, the hyperparameter controls the noise–resolution tradeoff in the reconstructed image. When the hyperparameter is too small, the resulting reconstruction will be noisy; when the hyperparameter is too large, the resulting reconstruction will be oversmoothed. In practice, this hyperparameter is determined empirically in a trial-and-error fashion for an optimal result, which is very time-consuming. In the past decades, several methods have been proposed to determine the optimal hyperparameter, such as the discrepancy principle [5], generalized cross validation [6], the L-curve method [7], the balance principle [8] and the Z-index method [9]. These methods directly or indirectly quantify the relationship between the image quality and the hyperparameter value. For example, the idea of the discrepancy principle is to select the hyperparameter so that the fidelity residual norm is close to a prior upper bound. Therefore, it requires the estimation of the noise level. The generalized cross-validation method is based on the hypothesis that a good hyperparameter should predict missing data values. It constructs an objective function of a hyperparameter and minimizes it. With the objective function, the L-curve plots the curve of fidelity residual norm vs. prior norm to determine the hyperparameter. Then, the optimal hyperparameter is at the corner of the “L” shape curve. The Z-index method plots the sparsity prior vs. the hyperparameter. The optimal parameter is selected at the second corner of the “Z” shape curve. Some iterative algorithms are also implemented to find the optimal hyperparameter [10,11]. However, all the aforementioned methods are conducted trial-and-error style and very time-consuming, even though the trials can sometimes be performed automatically by programs. To avoid this difficulty, a theory-based strategy is desired to determine the free hyperparameter, which can help to reduce the computational load.

Therefore, this study aims to investigate a theory-based strategy for parameter estimation in Bayesian image reconstruction without a freely adjustable hyperparameter. The main purpose of this study is the introduction of parameters to ensure that the condition of probability density function (PDF) in Bayes’ law is satisfied. Specifically, the Bayesian image reconstruction problem is formulated by two PDFs, one for the data fidelity term and the other for the prior term. Each PDF contains a parameter. While these two parameters ensure the PDFs completely describe the data and prior terms, they cannot be determined by the acquired data; thus, they are called complete but unobservable parameters. To differentiate from the conventional hyperparameter, we use the introduced parameters, which makes the PDF completely specified, and these are referred to as unobservable parameters hereafter. Estimating these two unobservable parameters becomes possible under the conditional expectation and maximization for the image reconstruction, given the acquired data and the PDFs. This leads to an iterative algorithm, which jointly estimates the unobservable parameters and computes the to-be reconstructed image by maximizing a posteriori probability (MAP), denoted as joint-parameter-Bayes. Although the joint Bayesian approach has been used in some studies [12,13,14], comprehensive studies are lacking that address the related issues, such as explanation, algorithm stability and so on. For instance, Hsiao et al. proposed one deterministic annealing method for transmission tomography based on Bayesian image reconstruction, where joint-MAP is used to estimate the parameters of mixed Gamma distribution in its prior model [12]. Cai et al. used joint-MAP to estimate the decomposition fractions and observation variance in Bayesian based dual-energy CT [13]. Zhang et al. applied this joint-MAP into cone beam X-ray luminescence computed tomography [14]. However, there are no extensive theory-based studies and discussions for parameter estimation in the mentioned studies. In this work, we investigate the stability of the proposed approach through factors including the initialization of the iterative process, the PDF specification, and renormalization in an iterative manner.

Additionally, by monitoring the unobservable parameters, another contribution of this work is to provide a sensitive metric to stop the iteration. Currently, most reconstruction terminates when the convergence criterion is met. It has already been reported that a better image can be obtained by stopping an iterative maximum likelihood algorithm early, well before convergence [15,16,17]. Veklerov and Llacer proposed a criterion based on a probabilistic interpretation [15,16]. Hebert reported that one explanation of the benefit of early termination is that the image degradation is one’s perception of the convergence process from the smooth initial image to the non-smooth maximum likelihood solution [17]. All these works inspired us to investigate the stopping criterion for this joint-parameter-Bayes method. The unobservable parameter of prior is found to have a turning point coincident with the optimal image quality in their curves along with iteration times. It is also found that the stopping criterion should be part of the joint-parameter-Bayes method. Ignoring the stopping criterion, however, may result in unsatisfying results.

Finally, given the acquired data, an optimized reconstructed image is obtained without any freely adjustable hyperparameters by satisfying the PDF condition for both data likelihood and a priori probability of Bayes’ law, and by satisfying the stopping criterion. Finally, although limited to the CT application in this paper, the proposed Bayesian image reconstruction can be generalized to other modalities and applications. This work is an extension of our previous conference paper of the 2020 IEEE Symposium on Nuclear Science [18].

## 2. Materials and Methods

### 2.1. Bayesian Image Reconstruction for CT

For an ill-posed problem such as low-dose CT (LdCT) image reconstruction, Bayesian reconstruction provides an alternative method for an improved solution compared to the deterministic method. According to Bayes’ law, we can have:

(1) $$p\left(\mu |y\right)=\frac{p(y|\mu )p\left(\mu \right)}{p\left(y\right)}\approx p\left(y|\mu \right)p\left(\mu \right)$$

where the vector $y=\{y_{i}|i=1,\ldots ,I\}\in {\mathbb{R}}^{I\times 1}$ represents the measured LdCT post-log sinogram data (with *I* data elements) and vector $\mu =\{\mu_{j}|j=1,\ldots ,J\}\in {\mathbb{R}}^{J\times 1}$ represents the linear attenuation coefficient distribution (with *J* image elements). The acquired data can be written as:

(2) $$y=\bar{y}+\zeta =A\mu +\zeta$$

where $\bar{y}\in {\mathbb{R}}^{I\times 1}$ is the expectation of y, $A=\left\{a_{ij}|i=1,\ldots ,I;j=1,\ldots ,J\right\}\in {\mathbb{R}}^{I\times J}$ is called the system matrix, and $\zeta \in {\mathbb{R}}^{I\times 1}$ indicates the noise vector corresponding to the measured sinogram data.

#### 2.1.1. Multivariate Gaussian Distribution Data Model

The post-log sinogram data of the ith X-ray, $y_{i}$, is the logarithm of transmission data $N_{i}$ and the incident photon flux $N_{0}$, which is calculated as:

(3) $$y_{i}=-log\;\left\{\frac{N_{i}}{N_{0}}\right\}.$$

In the transmission domain, the noise is usually modeled as the sum of Poisson and Gaussian distribution [19] as:

(4) $$N_{i}\;\sim \;Poisson\;\left\{{\bar{N}}_{i}\right\}+Gaussian\;\left\{m_{e},\;\sigma_{e}^{2}\right\}$$

where ${\bar{N}}_{i}$ is the mean transmission measurement of ith ray, and $m_{e}$ and $\sigma_{e}^{2}$ are the mean and variance value of the Gaussian type electronic noise. After logarithm, the non-linearity will be introduced, which can be approximated by Taylor expansion [20]. When only taking the leading order terms, the variance of post-log sinogram measurement is inversely proportional to the mean transmission data. Thus, the variance of ith sinogram measurement can be expressed as:

(5) $$Var\;\left(y_{i}\right)\;\propto \;1/{\bar{N}}_{i},$$

where ∝ is the proportional operator. Under the condition that all measurements $\left\{y_{i}\right\}$ are independent to each other, the covariance matrix is diagonal. If we denote the measured transmission counts as $\rho^{2}=\{\rho_{i}^{2}|i=1,\ldots ,I\}\in {\mathbb{R}}^{I\times 1}$, we have:

(6) $$\Sigma_{y}=diag\left\{1/{\bar{N}}_{i}\right\}=diag\left\{1/\rho_{i}^{2}\right\}$$

Given this knowledge, the PDF of the acquired sinogram can be expressed as:

(7) $$p\left(y|\mu ,s\right)=\overset{I}{\underset{i=1}{\prod }}\frac{1}{\sqrt{2\pi s/\rho_{i}^{2}}}exp\left\{-\frac{{\left(y_{i}-{\bar{y}}_{i}\right)}^{2}}{2s/\rho_{i}^{2}}\right\}.$$

where s is a parameter to completely define the variance.

#### 2.1.2. Multivariate Gaussian MRF Prior Model

While the linear attenuation coefficients are not random variables, their distribution patterns across the image can be assumed as random variables and the patterns are frequently modeled as the Markov random field (MRF). In this paper, the well-established Gaussian MRF penalty is used [21]. We assume that the variance of each pixel in the image field is $\sigma^{2}$, which is independent between two pixels. We can obtain the variance of the pair-wise pixel difference following the analysis for a pair-wise neighbor system as below:

(8) $$Var\left(\mu_{j}-\mu_{k}\right)=Var\left(\mu_{j}\right)+Var\left(\mu_{k}\right)-2Cov\left(\mu_{j},\mu_{k}\right)=\sigma^{2}+\sigma^{2}-0=2\sigma^{2}$$

where Var(.) represents the variance operator and Cov(.) denotes the covariance operator. If we denote $t=\sigma^{2}$, the Gaussian MRF *a priori* PDF can be expressed as:

(9) $$p\left(\mu |t\right)=\overset{J}{\underset{j=1}{\prod }}\frac{1}{\sqrt{2\pi t}}\;exp\left\{-\frac{\sum_{k\in \Omega_{j}}\omega_{jk}{\left(\mu_{j}-\mu_{k}\right)}^{2}}{2t}\right\},$$

where $\omega_{jk}$ represents the normalized pair-wise MRF coefficients among the neighbors $\Omega_{j}$ around the center *j*, $\underset{k\in \Omega_{j}}{\sum }\omega_{jk}=1$, and can be estimated based on some established knowledge or principles [22,23,24]. The scale factor is introduced as an unobservable parameter to ensure that the *a priori* PDF is completely specified for the pattern distribution to estimate the solution μ. One established MRF weight is used. In two-dimensional presentation, the eight image pixels weights in a 3 × 3 window can be given as:

(10) $$\left[\begin{array}{ccc} 0.104 & 0.146 & 0.104\\ 0.146 & 0 & 0.146\\ 0.104 & 0.146 & 0.104 \end{array}\right]$$

### 2.2. Unobservable Parameter Estimation by Joint-Parameter-Bayes

Introducing the PDF of the two terms of Equations (7) and (9) into Equation (1), we can obtain the solution by MAP estimation, expressed as:

(11) $${\hat{\mu }}_{MAP}=\mathrm{argmax}{|}_{\mu }\;\left\{\log p\left(\mu |y\right)\right\}\;\;\;\;\;\;\;\;\;\;\;\;\;\;\;\;\;\;\;\;\;\;\;\;\;\;\;\;\;\;\;\;\;\;\;=arg\;max{|}_{\mu }\;\left\{log\;p\left(\left(y|\mu \right)\right)+log\;p\left(\mu \right)\right\}\;\;\;\;\;\;\;\;\;\;\;\;\;\;\;\;\;\;\;\;\;\;\;\;\;\;\;\;\;\;\;\;\;\;\;\;\;\;\;\;\;\;\;\;\;=arg\;max{|}_{\mu }\;\;\left\{log\;p\left(\left(y|\mu ,s\;\right)\right)+log\;p(\mu |t)\right\}\;$$

However, the s,t are two unobservable parameters to completely obtain the PDF of data and penalty distribution. Therefore, for a given image μ, we use the joint-parameter-Bayes estimation of the two unobservable parameters. We can estimate s,t by:

(12) $$\hat{s}=arg\;max{|}_{s}\;\left\{log\;p\left(\left(y|\mu ,s\right)\right)\;\right\},$$

(13) $$\hat{t}=arg\;max{|}_{t}\;\;\left\{log\;p(\mu |t)\;\right\}.$$

According to the definition, for the *nth* iteration, with the current image estimate $\mu^{\left(n\right)}$, the data variance parameter ${\hat{s}}^{\left(n\right)}$ can be estimated by:

(14) $${\hat{s}}^{\left(n\right)}=\frac{1}{I}\overset{I}{\underset{i=1}{\sum }}\frac{1}{N_{i}}{\left(y_{i}-{\left[A\mu^{\left(n\right)}\right]}_{i}\right)}^{2}\;.$$

The prior variance parameter ${\hat{t}}^{\left(n\right)}$ can be obtained by:

(15) $${\hat{t}}^{\left(n\right)}=\frac{1}{J}\overset{J}{\underset{j=1}{\sum }}\underset{k\in \Omega_{j}}{\sum }\omega_{jk}{\left(\mu_{j}^{\left(n\right)}-\mu_{k}^{\left(n\right)}\right)}^{2}.$$

To solve the problem of Equation (11), we use the Newton method to iteratively find the solution. The pseudo code is summarized in Algorithm 1. For initialization, we first have the initial FBP image $\hat{\mu }$ from the measured post-log sinogram y. Then, a forward projection is applied on $\hat{\mu }$ with $q=A\;\hat{\mu }$. The residual $\hat{r}=y\;–\;q$ is obtained to describe the difference between estimation and measurement. $\rho^{2}$ describes the covariance factor obtained from measurement. The two unobservable parameters are initialized by Equations (14) and (15). For each iteration, we first update the attenuation map given the value of $\hat{s}$ and $\hat{t}$. Then, the residual r and the covariance factor $\rho^{2}$ are obtained based on the new attenuation map. Then, the $\hat{s}$ and $\hat{t}$ are updated orderly given the known attenuation. $A_{j}$ denotes the $j^{th}$ column of the system matrix A.
**Algorithm 1.** Joint-MAP-Bayes
***Initialization*:**
$\;\;\;\;\;\;\;\;\;\;\hat{\mu }=FBP\left\{y\right\}$$;\;q=A\;\hat{\mu }$$\;;\;\hat{r}=y\;–\;q$
$\;\;\;\;\;\;\;\;\;\;\rho^{2}=diag\left\{N_{0}e^{-q_{i}}\right\}\;$
$\;\;\;\;\;\;\;\;\;\;\lambda_{j}=A_{j}^{T}\rho^{-2}A_{j}$, ∀j
          Initialize $\hat{s}$ and $\hat{t}$ by Equations (14) and (15) with $\hat{\mu }$***For each iteration:******While (Stopping criterion is not met)***
*          For each voxel j:*
$\;\;\;\;\;\;\;\;\;\;{\hat{\mu }}_{j}^{old}={\hat{\mu }}_{j}$
$\;\;\;\;\;\;\;\;\;\;{\hat{\mu }}_{j}^{new}=\frac{A_{j}^{T}\rho^{-2}\hat{r}+\lambda_{j}{\hat{\mu }}_{j}^{old}+\frac{\hat{s}}{\hat{t}}\sum_{k\in \Omega_{j}}\omega_{jk}\mu_{k}}{\lambda_{j}+\frac{\hat{s}}{\hat{t}}}$
$\;\;\;\;\;\;\;\;\;\;{\hat{\mu }}_{j}=max\left\{0,\;{\hat{\mu }}_{j}^{new}\right\}$
$\;\;\;\;\;\;\;\;\;\;\hat{r}=\hat{r}+A_{j}\left({\hat{\mu }}_{j}^{old}-{\hat{\mu }}_{j}\right)$

          *end*

$\;\;\;\;\;\;\;\;\;\;\rho^{2}=diag\left\{N_{0}e^{-q_{i}}\right\}$
$\;\;\;\;\;\;\;\;\;\;\lambda_{j}=A_{j}^{T}\rho^{-2}A_{j}$
          Update $\hat{s}$ and $\hat{t}$ by Equations (14) and (15)

### 2.3. Stopping Criterion Investigation

According to the pseudo-code above, the stopping criterion is the only remaining part in our proposed framework. Conventionally, the mean squared difference between two successive reconstructed images is often employed as an indicator for the reconstruction convergency. It is also popular to assign a threshold of iteration times, e.g., 100 times based on experience. However, these methods always focus on the convergency from one indicator and pay little attention to image quality. As mentioned in the introduction, early stopping may provide better reconstruction results in the maximum likelihood algorithm. All these works inspired us to investigate the stopping criterion for this joint-parameter-Bayes method.

Based on the analysis from Section 2.2, we can see that there are two unobservable parameters in addition to the unknown attenuation map. Therefore, we can investigate the tendency of these two parameters along with their iterations. At the same time, we can quantitatively record the image quality, e.g., the mean squared error (MSE) compared to the ground truth. By doing so, we can investigate whether there is a correlation between the two parameters and image quality in the simulation, which always contains the ground truth. Based on this correlation, the real sinogram data experiment can also be evaluated.

### 2.4. Stability Investigation

**Figure 1:**
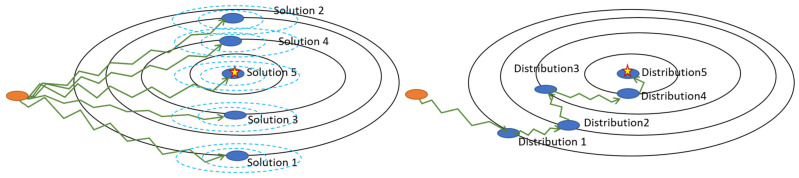
Comparison between the conventional MAP (**left**) and the proposed joint-parameter-Bayes (**right**).

Figure 1 compares the difference between the conventional MAP (left) and joint-parameter-Bayes (right). The orange region is one starting point. The star indicates the position of the optimal solution. For the conventional MAP method, we manually set one smoothness strength (hyperparameter) each time. After several trials, a set of solutions can be obtained. Then, the optimal solution is determined by comparing the final image quality. The proposed joint-parameter-Bayes method is more like that shown on the right hand. At every stage, it gives an estimation of the solution distribution and gradually approaches the optimal solution. Comparing the two methods, the conventional MAP is seen to be very time-consuming. The joint-parameter-Bayes method is much more convenient. However, the joint-parameter-Bayes method might give the “too far away” estimation and may lead to unsatisfying results. Therefore, the stability of the proposed joint-parameter-Bayes method should be explored from different factors, such as initials, variance and so on.

### 2.5. Phantom Simulation and Patient Data Acquisition

One advantage of using numerical phantom is that the simulation is fully controlled and the ground truth is available. Thus, we used the classical Shepp–Logan phantom to explore the effectiveness and stability of the proposed joint-parameter-Bayes method. The Shepp–Logan phantom includes 512 × 512 pixels with a resolution of 0.74 mm × 0.74 mm. This simulation setup is the same as that for the clinical patient data used in this work. Specifically, the distance from the source to the system center was 570 mm, and the distance from the source to the detector was 1040 mm. The sinogram data has 672 detector elements with a width of 1.4 mm per element, and 1160 projection views over a 360° range by forward projection. A clinical patient was also recruited to this study under informed consent after approval by the Institutional Review Board (IRB). The patient was scanned with normal dose settings with a tube voltage of 120 kVp and a tube current of 100 mAs. The geometric settings are consistent with the simulation study. Since the sinogram is from a normal-dose scan, we just used the reconstruction by FBP as reference for comparison.

## 3. Results

### 3.1. Results of Numerical Simulation Data

#### 3.1.1. Reconstruction Comparison

In Section 2.1, the multivariate Gaussian distribution data model is described in detail. Note that this variance model analysis is based on the realistic CT signal generation model, i.e., the compound of the Gaussian and Poisson model. In a simplified simulation case, the variance can be degenerated to be a scaler when directly applying white Gaussian noise to the post-log sinogram. With simulations, we can manipulate the noise type to perform comprehensive evaluations.

The simple noise model is used to add the white Gaussian noise directly to the post-log sinogram data. This provides a prefect evaluation model, since we know exactly the value of s. If we add noise $\zeta \sim Gaussian\left(0,\;\sigma_{G}^{2}\right)$, s will be of the value $\sigma_{G}^{2}$ by assigning $\rho_{i}^{2}=1$. We can evaluate the estimation of $\hat{s}$ by the proposed method. In the experiment, five noise levels are simulated with $\sigma_{G}^{2}=\left\{0.1^{2},0.3^{2},0.5^{2},0.7^{2},0.9^{2},1.1^{2}\right\}$. For a comparison of the reconstructed images, the conventional MAP method is regarded as the baseline. For the baseline, we swept its hyperparameter within a wide range by sampling three points at each order of magnitude. For each sampled hyperparameter, the reconstruction is stopped after 1000 iterations. If we denote the reconstruction for one sampled hyperparameter as one trial, the computing time for the conventional method is $Time_{MAP}=time/iteration\;*\#iterations*\;trial\;number$. It is ensured that the reconstructed images are from too noisy to oversmoothed. Visual judgement and quantitative measures are used to determine the optimal images as the results of the baseline.

**Figure 2:**
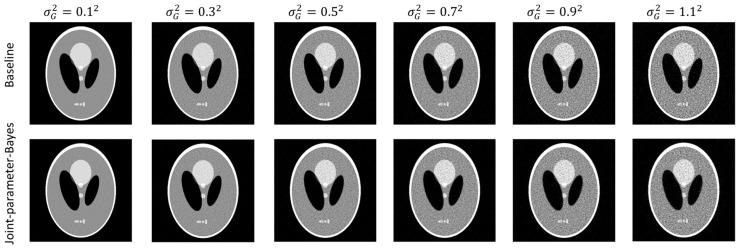
Comparison of reconstructed images between the conventional MAP (**top row**) and joint-parameter-Bayes (**bottom row**). The display window is [0, 0.035] mm^−1^.

Figure 2 shows the reconstructed images both by the conventional MAP (baseline) and joint-parameter-Bayes with different noise levels. It can be observed that the results are comparable. The quantitative comparisons are summarized in Table 1 which are also consistent with our visual inspection. However, their computation time is quite different based on the iterations they use in Table 1. The proposed joint-parameter-Bayes saves approximately 66~88% of computation time in comparison with the baseline method. The number of iterations for joint-parameter-Bayes is further discussed in the stopping criterion subsection. To test the robustness, we performed the experiments on two other noise types: (1) Poisson-like signal-dependent noise to post-log sinogram data; (2) Poisson noise to the pre-log transmission data. We repeated the above evaluations and yielded very similar results, which can be found in the Appendix A. Some details of the two noise models are also be discussed in the following subsections.

**Figure 3:**
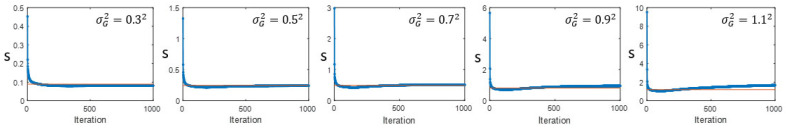
Comparison of estimated s by the joint-parameter-Bayes method (blue curve) and the true value of s (red curve). They agree well with each other, demonstrating the effectiveness of our proposed estimation method.

To evaluate the estimation of joint-parameter-Bayes for the unobservable parameters, we also recorded the value of s with the iteration times for a different simulation. The results are shown in Figure 3. The red line is the simulated value or true value of s. The blue curve is the estimated value by the joint-parameter-Bayes method. It is found that the estimated s agrees well with the true value, which demonstrates the effectiveness of the estimation method. It is also noted there is slightly higher disagreement when the iteration passes a certain number, especially in the high-noise scenarios, e.g., $\sigma_{G}^{2}=1.1^{2}$. This observation also indicates that it might be better to terminate the iteration early.

#### 3.1.2. Stopping Criterion Investigation

As described in Section 2.3, the stopping position (i.e., different number of iterations) may result in different image quality. In this section, we investigate the stopping criterion for the proposed joint-parameter-Bayes by exploring the correlation between the two unobservable parameters and image quality. To see the response of the stopping criterion to different noises, three noise types mentioned above are added to the simulation data, including white Gaussian noise to post-log sinogram data, Poisson-like signal-dependent noise to post-log sinogram data, and Poisson noise to the pre-log transmission data. As introduced in Section 3.1.1, we can manipulate the variance $\sigma_{G}^{2}$ to mimic different noise levels for white Gaussian noise. For Poisson noise to sinogram data, we scale the values of Shepp–Logan phantom by different factors and then add Poisson noise to its sinogram data from forward projection. By doing so, we can mimic different noise levels by varying the factor value. Three scaling factors are used, which are 1, 5 and 10. For the Poisson noise to transmission data, it is more like the realistic noise model introduced in Section 2.1.1. By varying the incident flux $N_{0}$, we simulated different noise levels. In this work, we chose five incident flux, $N_{0}=\left\{1\times 10^{4},5\times 10^{4},1\times 10^{5},5\times 10^{5},1\times 10^{6}\right\}$.

**Figure 4:**
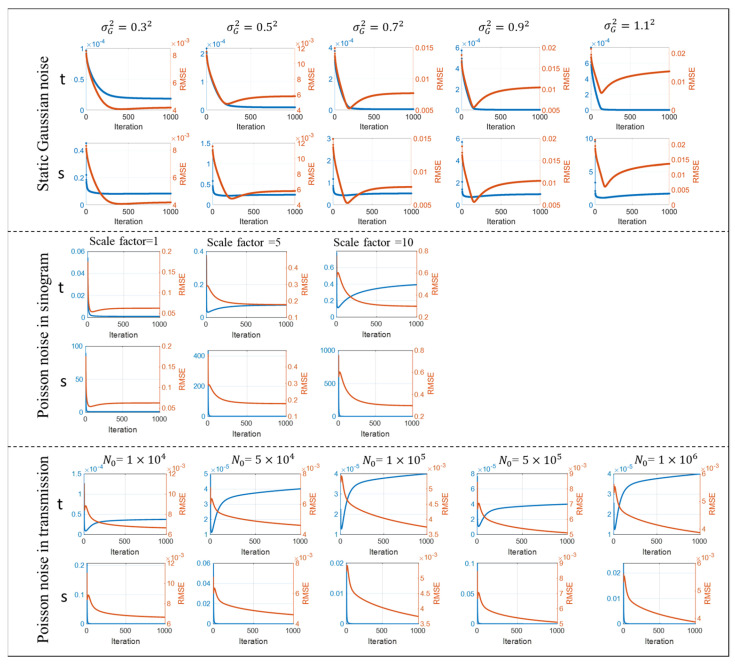
Stopping criterion under three different noise types. The change of two unobservable parameters (s, t) and change of image quality (RMSE) along with iteration are plotted.

Figure 4 shows the change of two unobservable parameters along with their iteration. The image quality measure RMSE is also plotted in the same figure to find some correlations. It can be found the unobservable parameter t has a coincident turning point with the optimal image quality. For example, in the case of $\sigma_{G}^{2}=0.7^{2}$, the image quality (red line) has a valley around 250 iterations. The s curve has a very fast drop at several iterations and then becomes flat. The t curve has a relatively slow drop and then becomes flat. If we call the largest slope rate change point the turning point, the turning point of image quality and t are almost identical. Therefore, we can monitor the transformation of t along with iteration and terminate the reconstruction at its turning point, since the image quality measure RMSE cannot be obtained in real cases. For the other two noise types, the above observations are found in the case of scale factor = 1. The change of s remains the same, but the t curve decreases first and then increases. Interestingly, the image quality also deteriorates first and then improves. After the changing point, both image quality and t tend to converge. One possible reason for the initial un-monotonic tendency could be the initial condition. This effect can be removed, however, if we start to monitor the value of t several iterations (e.g., 10 iterations) late. After the removal of the initial effect, the regulations can be summarized as if t decreases monotonically, the reconstruction can stop at the turning point, and if t increases, the reconstruction can stop when it is converged. It is interesting to find that the prior variance parameter t has such an explicit correlation with the image quality. Data variance parameter s has no such clear tendency. One possibility could be that the prior variance t is directly related to the image domain and data variance parameter s is directly related to the measurement. Therefore, the relationship between image quality and t is not explicit. It is also noted that the t curves have different patterns under different noise conditions, regardless of the initial effect. This is expected because the data-independent, data-dependent and logarithm non-linearity type noises are introduced on purpose. The three noise types cover the most popular models used in the image reconstruction. Based on the observation, we find some semi-experimental regulation of the early stop criteria for optimal image reconstruction. To fully understand the curve patterns under different noise conditions is one of our future research interests.

**Figure 5:**
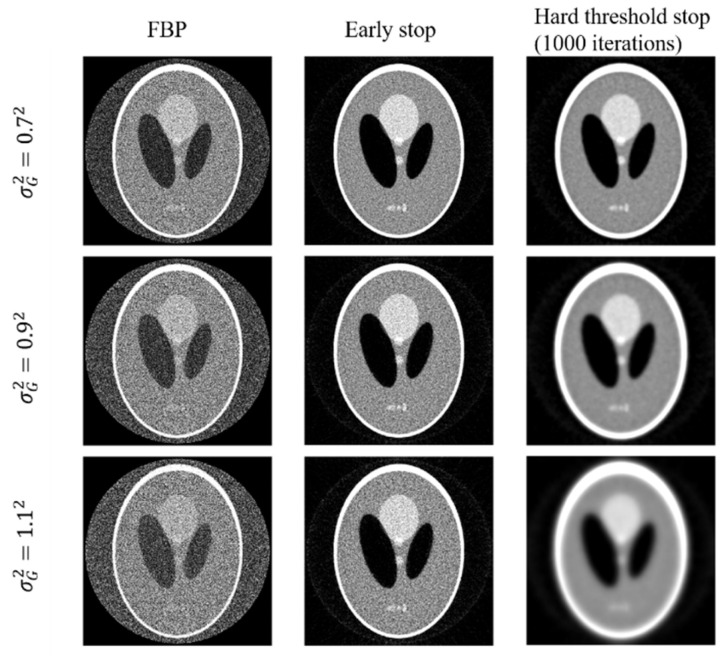
Reconstructed images by FBP (**left column**) and the joint-parameter-Bayes method with early stop (**middle column**) and hard threshold stop (**right column**). The display window is [0, 0.035] mm^−1^.

In the experiments, it is noticed that the stopping criterion should be part of the joint-parameter-Bayes method. Ignoring the stopping criterion may result in unsatisfying results. We presented the reconstructed images of early stop criterion and hard threshold in Figure 5. We can see that the early stop can produce better reconstructed images.

#### 3.1.3. Stability Investigation

(a) Effect of Initialization

**Figure 6:**
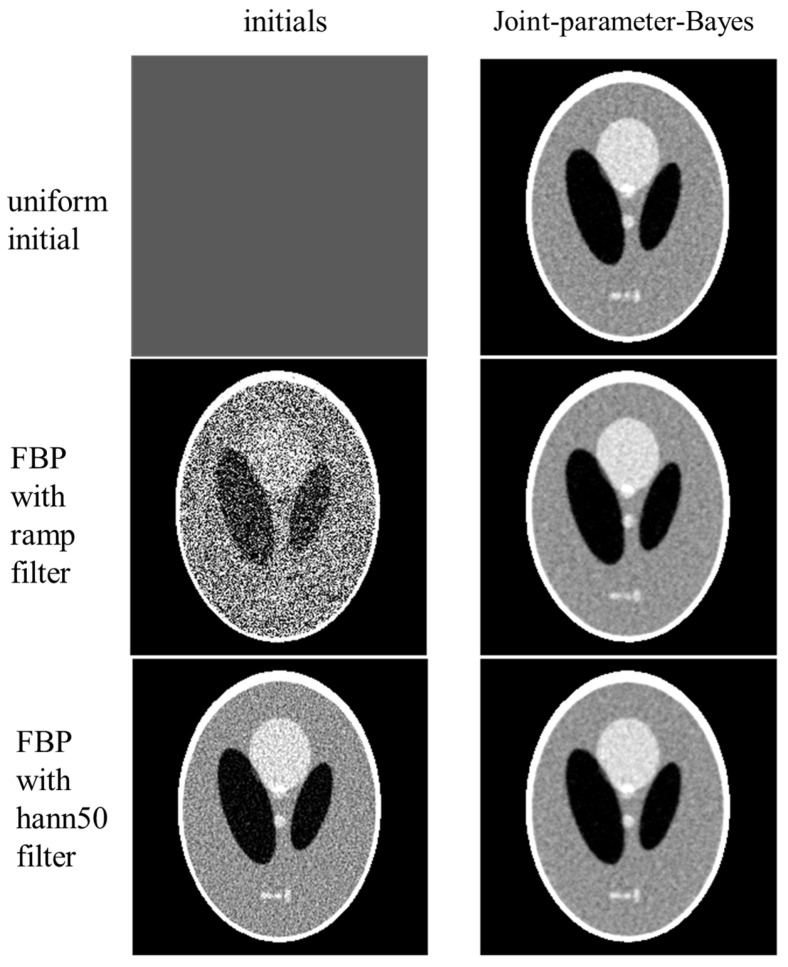
Performance of joint-parameter-Bayes with different initials. From top to bottom, the initials (**left column**) are uniform averaged from FBP, FBP with ramp filter, and FBP with hann50 filter, while the corresponding joint-parameter-Bayes reconstructions are shown on the right (**right column**).

**Figure 7:**
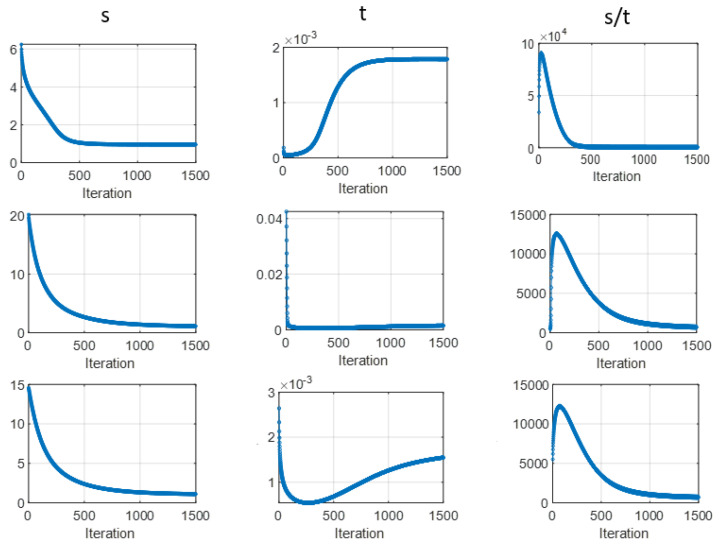
Estimated free parameters by joint-parameter-Bayes along with iterations with three different initials in accordance with Figure 6.

We further investigated the effect of initialization on the proposed joint-parameter-Bayes method. Three different initials were tested: uniform initial by averaging the FBP results, FBP with ramp filter, and FBP with hann50 filter. We investigated the effect on the Poisson-like signal-dependent noise to post-log sinogram data. This simulation provides the opportunity to deal with the signal-dependent noise data, where the signal variance is known to equal its signal mean. This means that the true value of $\rho^{2}$ is y. First, we used the true variance y for $\rho^{2}$, instead of the estimated $\hat{y}$ and just varied the initials. The reconstructed images and their initials are presented in Figure 6. The estimated parameters along the iterations are presented in Figure 7. According to Figure 7, the pattern of s does not change too much for different initials, but the pattern of t varies. Their ratio s/t follows a similar pattern with a different convergence rate. According to Figure 6, the reconstructed results are stable with all three different initials. This implies that joint-parameter-Bayes can effectively estimate the parameter values starting with different initials and provide stable solutions anyway when knowing the true variance.

**Figure 8:**
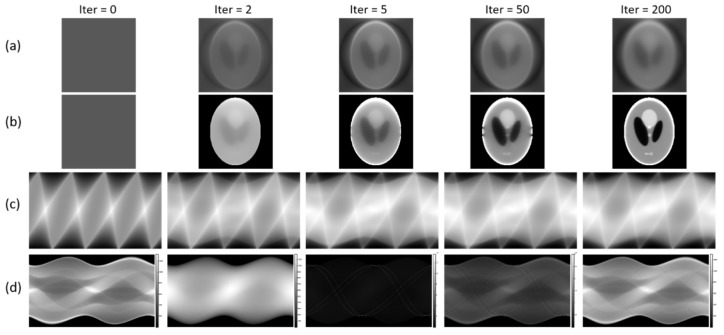
(**a**,**b**): Reconstructed images at different iterations with uniform initials but different initial variance. (**c**,**d**): The change of variance $\rho^{2}$ at different iterations according to (**a**,**b**).

We then tested the initials by only knowing that the variance distribution is Poisson. Specifically, $\rho^{2}$ is estimated by the $A\hat{\mu }$ and updates every iteration. Stable reconstructions are still observed with FBP initials. However, something interesting is observed for the uniform initials. Figure 8a presents the reconstructed images at different iterations with the same uniform initial used in Figure 6. Comparing Figure 6 and Figure 8a, the only difference is the variance value $\rho^{2}$. Therefore, we recorded the change of $\rho^{2}$ estimated by $A\hat{\mu }$ at different iterations, shown as Figure 8c. In the simulation, we know that the ground truth of variance should have the pattern of the first image in Figure 8d. However, the estimated variance in Figure 8c cannot produce the right estimation. To further investigate the effect, we directly use the ground truth variance as the initial variance and still use $A\hat{\mu }$ to estimate the variance for the following iterations. The tracked reconstructions and variance estimation are shown Figure 8b,d. According to Figure 8b, joint-parameter-Bayes can somehow give us a reasonable reconstruction. From Figure 8d, although the variance is not estimated very accurately during the iteration, finally, it can return by giving a good initial. Therefore, this joint-parameter-Bayes depends on the initial values of the parameters. In the experiments, it is found that the initials using FBP are quite robust, and they are recommended for joint-parameter-Bayes.

(b) Effects of Variance Normalization

**Figure 9:**
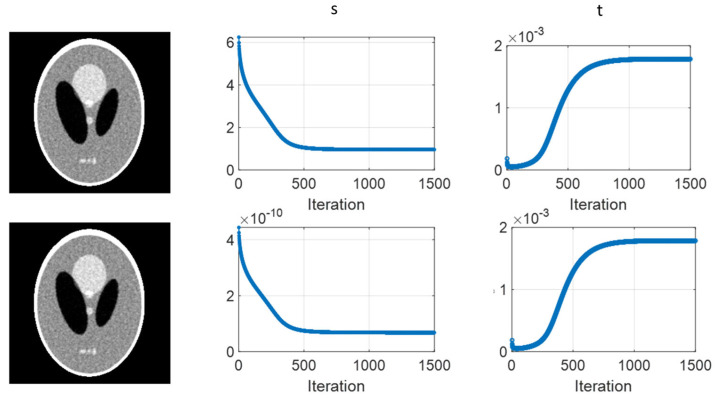
Performance of joint-parameter-Bayes images for $\rho^{2}$ without (**upper**) and with normalization (**lower**) by $sum\left(\rho^{2}\right)=1$. The CT image display window is [0, 0.035] mm^−1^.

As we know, $\rho^{2}$ is the variance factor from the measurement. The element value range might vary from different modalities. For example, in the simulation, $\rho_{i}^{2}=y_{i}$ for the Poisson-like signal-dependent noise to post-log sinogram data, and $\rho_{i}^{2}=N_{i}$ for the Poisson noise to the pre-log transmission data. Therefore, it is useful to know whether the normalization of matrix $\rho^{2}$ will affect the performance of the joint-parameter-Bayes method. Figure 9 presents the reconstructed images for $\rho^{2}$ without (upper) and with normalization (lower) by $sum\left(\rho^{2}\right)=1$ on the Poisson-like signal-dependent noise to post-log sinogram data. The scale factor is 1. Comparable reconstructed images are obtained. The estimated parameters follow the same pattern with linear scaling strength. This observation agrees with our expectation that even though the magnitude of s and t are scaled due to normalization, the fidelity strength and prior strength will also be scaled by the same degree. Therefore, the results would not be affected.

### 3.2. Results of Clinical Patient Data

**Figure 10:**
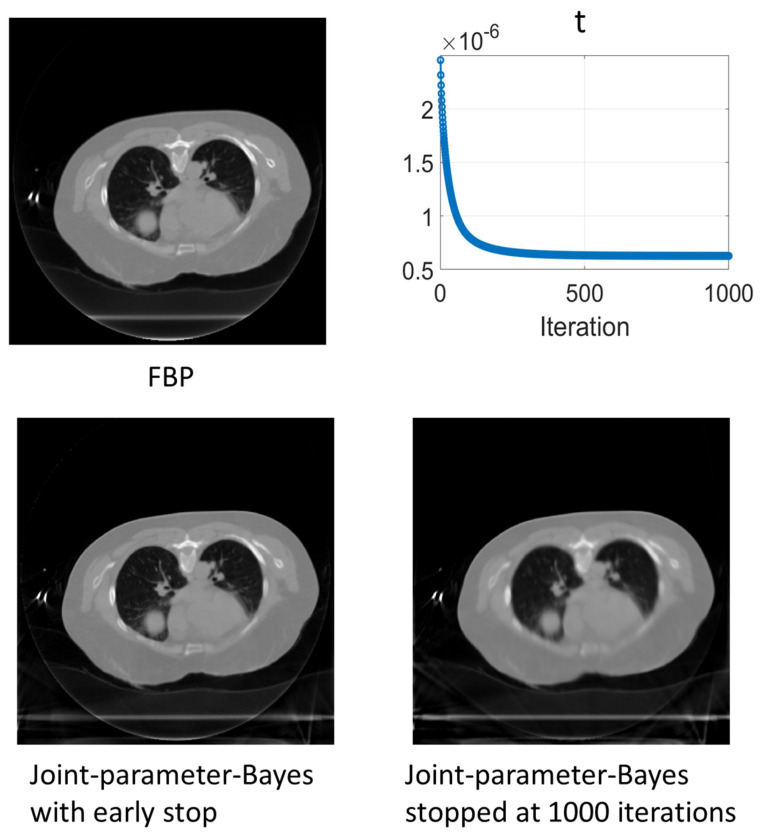
Comparison of reconstructed images between the conventional MAP and joint-parameter-Bayes. The CT image display window is [0, 0.035] mm^−1^.

Figure 10 shows the results of the reconstructed images using different algorithms. The pattern of t along with iteration is also shown in the top right panel. The early stopping criterion used is the turning point of t, as described in Section 3.1.2. From the t curve, we can see that it has a rapid decrease and then becomes flat. According to the experimental study in Section 3.1.2, for this type of t curve pattern, the turning point of t—which is the largest slope rate change point—will give the best image quality. This reconstruction should stop early at this iteration number. The reconstruction with early stopping criteria is shown in the bottom left panel. For comparison, the reconstruction with 1000 iterations is shown in the bottom right panel. According to Figure 10, joint-parameter-Bayes can give a comparable image with the reference using visual judgement. With the convergence stopping criterion, the reconstructed image of joint-parameter-Bayes is blurred. Early stopping is necessary to obtain satisfying results. The stopping criterion obtained from the simulation model has also shown its effectiveness in clinical patient data.

## 4. Discussion

We performed extensive studies to investigate the joint-parameter-Bayes method without a freely adjustable hyperparameter. It is found that an early stopping criterion is necessary for optimal image quality. The stopping criterion constructed from one of the unobservable parameters has shown its effectiveness and robustness through three different noise models and evaluation on the clinical patient data. However, this criterion observed is based on the initial image being FBP-ramp. More evaluations are needed to investigate the stopping criterion under different conditions. Currently, we construct the criterion based on our observations in the experiments. It is still unclear why the two unobservable parameters have the patterns they do and why the parameter of the prior is more sensitive.

In the conventional MAP method, the hyperparameter is used to balance the data fidelity term and the prior/penalty term, which is a constant through the reconstruction process. Comparing the updating equation of the attenuation map between joint-parameter-Bayes (see the pseudo code) and the conventional MAP method [23], we can see that the hyperparameter of the conventional method is proportional to the ratio of unobservable parameters s and t, where there might be some scale factor considering the normalization effect. As we know, s is introduced to completely specify the data fidelity PDF, and t is introduced to completely specify the prior term PDF. s is related to the data fidelity variance and t is related to the prior variance. This might be one interpretation of the physical meaning of the hyperparameter, which is related to the ratio of two unobservable parameters reflecting the data fidelity variance and the prior variance. This implies that, given the count-limited imaging, such as LdCT, we desire high confidence or low variance on the prior penalty. In other words, we desire the medical truth to derive prior and train machine learning. Additionally, in the reconstruction of joint-parameter-Bayes, the s/t is no longer a constant, which will be self-adjusted by the current reconstruction estimation. In the future, we will compare the proposed method with the deep learning-based approaches of estimating the optimal hyperparameter for the MAP methods.

There is another remaining issue to be addressed. This work used the normal-dose clinical patient data to evaluate the performance of the joint-parameter-Bayes algorithm. In the future, evaluations on the LdCT and other modalities are needed to validate the model.

## 5. Conclusions

A theory-based joint-parameter-Bayes method is presented in this work to avoid the freely adjustable hyperparameter for penalized maximum likelihood tomographic image reconstruction. The strategy is based on the PDF requirements of Bayes’ law. For both fidelity and prior term in Bayesian reconstruction, their multivariate Gaussian PDF distributions are completely described by two unobservable parameters, which are jointly estimated by maximizing a posteriori probability for the proposed Bayesian reconstruction. The optimal image can be reconstructed by joint-parameter-Bayes without any freely adjustable hyperparameter for the given data, by satisfying the PDF condition for both the data likelihood and the prior probability of Bayes’ law, and by satisfying the stopping criterion. Compared to the conventional MAP method, the proposed scheme can save at least 66% computation time. It has great potential for application to joint-parameter-Bayes to improve the reconstructed image quality with incomplete data, such as LdCT and limited angle CT. It can also be generalized to other tomographic modalities and applications, such as positron emission tomography and ultrasound imaging reconstruction.

## Figures and Tables

**Table 1:** Quantitative comparison of the conventional MAP (baseline) and joint-parameter-Bayes.

Conventional MAP (Baseline)					Joint-Parameter-Bayes			
$\sigma_{G}^{2}$	RMSE	SSIM	PSNR	Iterations	RMSE	SSIM	PSNR	Iterations
$0.1^{2}$	0.002397	0.9970	52.41	1000 * trial number	0.002264	0.9972	52.90	1000
$0.3^{2}$	0.003581	0.9911	48.92	1000 * trial number	0.004125	0.9918	47.69	339
$0.5^{2}$	0.004440	0.9894	47.05	1000 * trial number	0.004778	0.9881	46.42	200
$0.7^{2}$	0.004995	0.9867	46.03	1000 * trial number	0.00518	0.9853	45.71	157
$0.9^{2}$	0.005190	0.9844	45.67	1000 * trial number	0.005501	0.9829	45.19	132
$1.1^{2}$	0.005426	0.9815	45.31	1000 * trial number	0.005786	0.9805	44.75	115

**Figure A1:**
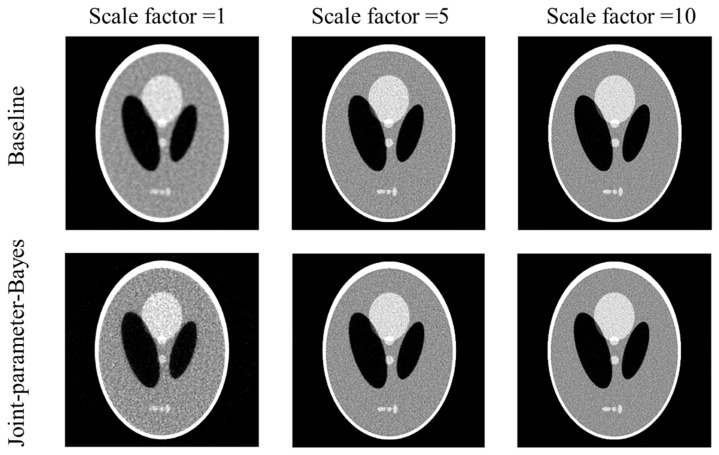
Comparison of reconstructed images between the conventional MAP (baseline) and joint-parameter-Bayes with Poisson noise to post-log sinogram data.

**Figure A2:**
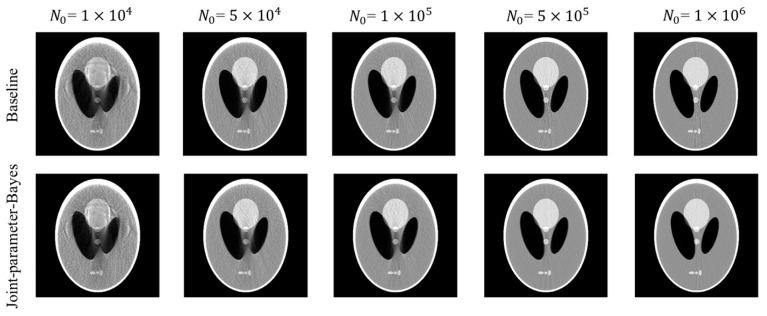
Comparison of reconstructed images between the conventional MAP (baseline) and joint-parameter-Bayes with Poisson noise to pre-log transmission data.

## Data Availability

Patient sinogram data is unavailable due to privacy or ethical restrictions.

## Appendix A

Figure A1 and Table A1 present the comparison results between the baseline and the proposed joint-parameter-Bayes on Poisson noise in post-log sinogram data. Figure A2 and Table A2 present the comparison results between the baseline and the proposed joint-parameter-Bayes on Poisson noise to pre-log transmission data.

**Table A1:** Quantitative comparison of joint-parameter-Bayes with conventional MAP (baseline) with Poisson noise to post-log sinogram data.

Conventional MAP (Baseline)					Joint-Parameter-Bayes			
Scale Factor	RMSE	SSIM	PSNR	Iterations	RMSE	SSIM	PSNR	Iterations
1	0.0643	0.9109	23.84	1000 * trial number	0.0547	0.8249	25.24	81
5	0.1844	0.6445	14.68	1000 * trial number	0.1848	0.6358	14.67	1000
10	0.2994	0.6091	10.48	1000 * trial number	0.3073	0.5904	10.25	1000

**Table A2:** Quantitative comparison of joint-parameter-Bayes with conventional MAP (baseline) with Poisson noise to pre-log transmission data.

Conventional MAP (Baseline)					Joint-Parameter-Bayes			
Incident Flux	RMSE	SSIM	PSNR	Iterations	RMSE	SSIM	PSNR	Iterations
$1\times 10^{4}$	0.0065	0.9744	43.69	1000 * trial number	0.0067	0.9731	43.53	1000
$5\times 10^{4}$	0.0048	0.9864	46.36	1000 * trial number	0.0051	0.9853	45.83	1000
$1\times 10^{5}$	0.0042	0.9898	47.46	1000 * trial number	0.0046	0.9885	46.73	1000
$5\times 10^{5}$	0.0033	0.9941	49.68	1000 * trial number	0.0038	0.9931	48.25	1000
$1\times 10^{6}$	0.0031	0.9946	50.04	1000 * trial number	0.0037	0.9937	48.53	1000
